# A cross-sectional survey on availability of facilities to healthcare workers in Pakistan during the COVID-19 pandemic

**DOI:** 10.1016/j.amsu.2020.09.027

**Published:** 2020-09-23

**Authors:** Adeel Abbas Dhahri, Muhammad Rafaih Iqbal, Abul Fazal Ali Khan

**Affiliations:** a The Princess Alexandra Hospital NHS Trust, Hamstel Road, Harlow, CM20 1QX, United Kingdom; b Khuda Bux Dhahri Clinic, Tando Agha Hyderabad, 71000, Sindh, Pakistan; c Mid City Hospital, 3-A Shadman II, Jail Road, Lahore, Pakistan

**Keywords:** COVID-19, Doctors, Nurses, Personal protective equipment (PPE), Pakistan

## Abstract

**Introduction:**

COVID-19 pandemic has caused a healthcare crisis across the world. Low-economic countries like Pakistan lag behind in an adequate response including supply of Personal Protective Equipment (PPE), leading to panic among healthcare workers. We aim to evaluate hospital settings and state in Pakistan regarding availability of resources and views of healthcare workers on COVID-19.

**Method:**

A questionnaire survey was carried out among healthcare workers in public and private sector hospitals across Pakistan for a period of one month. The primary measured outcomes were presence of local Standard Operating Procedures (SOPs), availability and training of PPE, specific isolation wards and staff wellbeing support by the hospital management.

**Results:**

There were 337 participants, 307 (91.1%) doctors and 11 nurses (3.3%). About two-third of the participants (n = 199, 59%) reported non-availability of PPE and 40% (n = 136) denied availability of local Standard Operating Procedures. About a quarter of the participants (n = 94, 27.8%) had training in Donning and Doffing. Most of the participants (n = 277, 82.1%) felt that it was necessary to have testing available for frontline workers.

**Conclusion:**

There is lack of PPE and adequate facilities in hospitals as COVID-19 continue to spread in Pakistan. Local medical governing bodies and societies should come forward with guidelines to ascertain wellbeing of the healthcare workers.

## Highlights

• This is a survey of healthcare professionals in Pakistan on the availability of facilities during the peak of covid 19.
• Two-third of the participants reported non-availability of PPE.
• Only a quarter of the participants reported training in Donning and Doffing.

## 1 Introduction

World Health Organization (WHO) declared COVID-19 outbreak as a Public Health Emergency of International Concern on January 30, 2020 after proven human-to-human transmission [1]. Pakistan, sharing its borders with highly affected countries, reported its first case in late February 2020 and since then, COVID-19 continue to make its way throughout the country [2]. According to Official Pakistan Government statistics as of 12th September, more than 300,955 cases have been confirmed with total number of deaths exceeding 6373 [3].

Healthcare workers are at risk of repeated exposure because patients are referred to hospitals or isolation centers for diagnosis, quarantine and management [4]. Implementation of infection prevention and control is of great significance and an increase in awareness of transmission of COVID-19 infection, self-isolation and personal protection plays an important role in reducing the risk of infection to healthcare workers. As the world continue to learn from COVID-19, National and International guidelines on Personal Protective Equipment (PPE) continue to evolve [3,[5], [6], [7]].

PPE is defined as equipment that protects healthcare worker against infection risks at work and its use is not new in prevention of infection. It consists of gloves, mask, face shield, gown, head cover and rubber boots [8,9]. While there have been reports of transmission of COVID-19 during the incubation period in different parts of the world, a sense of panic continues to grow among healthcare workers in Pakistan as the number of daily positive cases continue to increase along with the mortality [10,11]. As the demands increased due to use and supply strategies of PPE, worldwide shortages have been reported and government have been struggling to satisfy the demands [12,13].

Different reasons have been described as a cause of COVID-19 spread among healthcare workers including inadequate availability of PPE due to international shortage, lack of knowledge, inadequate training, intensity of work and long-time exposure to infected patients. Another concept among healthcare workers is to protect their health by expanding COVID-19 screening tests among them in order to reduce the risk of transmission [14,15].

We aim to assess the hospital situation and opinion of healthcare workers in Pakistan on COVID-19.

## 2 Method

We conducted a prospective 14-question survey of healthcare workers across Pakistan from April 15, 2020 to May 15, 2020. The questionnaire utilized Google Forms and was distributed through WhatsApp with all questions mandatory. The questionnaire was based on Likert-item scale. Healthcare workers were defined as all the hospital staff members working in the hospital, including doctors, nurses, healthcare assistances, physiotherapists, pharmacists, managers and cleaning staffs.

The survey included questions regarding the province of work, type of hospital (public/private/both) and role in the hospital. Hospital situation was assessed by asking presence of local Standard Operating Procedures (SOPs), availability of disposable face masks, availability of PPE, training in Donning and Doffing, specific isolation wards and helpfulness of the hospital management. Further questions included source of information regarding COVID-19 and if it has improved hand washing. We also asked participants’ views on preparation of Pakistan for COVID-19 like strengthening systems and capacity, engagement of the societies for effective response. They were also asked about social lockdown and COVID-19 swab or antibody testing for healthcare workers.

All participants voluntarily participated in this study and were informed that the information they provide will remain confidential and will not be used to identify individual replies. Questionnaire survey is available in the supplementary information.

The study has been reported in line with the STROCSS criteria [16]. The study has also been registered at http://www.researchregistry.com and the unique identifying number is: researchregistry5884 [17].

As this survey focused on healthcare workers who volunteered themselves in anonymized online survey so ethical approval was not required.

## 3 Results

A total of 337 volunteers participated in this survey from all over Pakistan. Majority of the participants were from Sindh (n = 207, 61.4%) followed by Punjab (n = 122, 36.2%). Three (0.9%) participants were from Baluchistan, two (0.6%) from Khyber Pakhtunkhwa, while three (0.9%) from Gilgit Baltistan. Participants mostly included doctors (n = 307, 91.1%), nurses (n = 11, 3.3%) and managers (n = 11, 3.3%) (Table 1). Most of the participants were from public sector hospitals (n = 188, 55.8%) followed by private ones (n = 89, 26.4%) while 17.8% (n = 60) were working in both public and private hospitals.

**Table 1:** Role of the participants in the Hospital.

Role	n, %
Doctor	307, 91.1
Nurse	11, 3.3
Healthcare Assistance	2, 0.6
Manager	11, 3.3
Pharmacist	1, 0.3
Physiotherapist	2, 0.6
Other	3, 0.9

A total of 126 (37.3%) participants said that they had SOPs regarding COVID-19 at their hospital while 136 (40.3%) denied it. 78% (n = 263) of the participants reported that they had disposable face masks at their hospital while 19.8% (n = 67) denied it. With regards to the availability of PPE, 59% (n = 199) responded that it was not available while only 24.9% (n = 84) were happy with its availability at their hospital. When questioned about training in Donning and Doffing, 56.9% (n = 192) had received no training while only 27.8% (n = 94) said that they had appropriate training. About one third of the participants (n = 113, 33.5%) said that there were specific isolation wards in their hospital for COVID-19 patients and nearly the same (n = 111, 32.9%) reported that the hospital management was not helpful to them (Table 2).

**Table 2:** Hospital preparation details (n,%).

	Strongly agree	Agree	Neither agree nor disagree	Disagree	Strongly disagree
SOPs	27, 8	99, 29.4	75, 22.3	102, 30.3	34, 10.1
Face masks	199, 59	64, 18.9	31, 9.1	32, 9.4	11, 3.2
PPE	21, 6.2	63, 18.7	54, 16	116, 34.4	83, 24.6
Training	18, 5.3	76, 22.6	51, 15.1	136, 40.4	56, 16.6
Isolation ward	28, 8.3	85, 25.2	69, 20.5	105, 31.2	50, 14.8
Helpful management	40, 11.9	107, 31.8	79, 23.4	74, 22	37, 11

SOP: Standard Operating Procedure, PPE: Personal Protective Equipment.

**Fig. 1:**
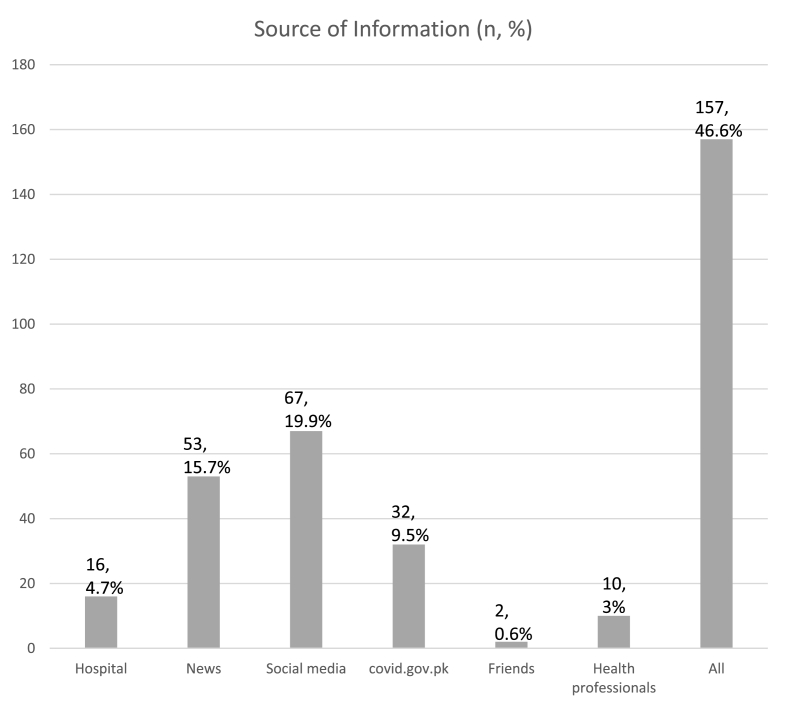
Source of information regarding COVID-19.

Approximately half of the participants (n = 157, 46.6%) stated that their source of information about COVID-19 were multiple including news, social media, Pakistan Government website and hospital based (Fig. 1). The vast majority of participants (n = 310, 91.9%) reported that their hand-washing practice has improved since COVID-19 pandemic started (Table 3).

**Table 3:** Improved hand washing.

	n, %
Strongly agree	139, 41.2
Agree	171, 50.7
Neither	22, 6.5
Disagree	5, 1.5
Strongly disagree	0, 0

Most of the participants (n = 220, 65.2%) were not happy with the preparations. Similarly, majority (n = 282, 83.6%) were in favour of strict lockdown rather than smart lockdown and testing the healthcare workers (n = 277, 82.1%) for COVID-19 (Table 4).

**Table 4:** General views (n,%).

	Strongly agree	Agree	Neither agree nor disagree	Disagree	Strongly disagree
Pakistan preparation	3, 0.9	43, 12.8	71, 21.1	146, 43.3	74, 22
Strict Lockdown	143, 42.4	139, 41.2	36, 10.7	16, 4.7	3, 0.9
Testing for HCWs	180, 53.4	97, 28.8	24, 7.1	30, 8.9	6, 1.8

(HCWs: healthcare workers).

## 4 Discussion

COVID-19, since its origin in Wuhan has spread at a rapid pace all over the world. It poses a great threat to us as our generation has not witnessed a pandemic like this before [18]. Different countries are currently working on COVID-19 vaccine with variable results being gathered to find a safe and effective vaccine [19,20]. Almost all of the countries have been surprised with this pandemic and are struggling to cope with the disastrous effects of it. Management of such pandemic revolves around slowing the spread the disease also called as flattening the curve so that the healthcare systems are not overwhelmed [1].

Due to the increasing number of patients affected, the healthcare systems are under tremendous strain and there have been issues with the availability of PPE globally, even in the most advanced countries either due to the non-availability of it or due to imbalance between demand and supply [12].

Our study showed that only 24.9% of the healthcare workers were happy with the availability of PPE at their hospital. It is understandable in a low economic country like Pakistan but this issue is global. A similar study conducted in UK also showed that even there they had difficulties in receiving adequate PPE [21,22]. As the COVID-19 makes its way through to the world, the demand for PPEs has increased significantly and many developed countries are also facing this challenge [19]. The safety of healthcare workers is as important as the safety of the patient. Not only employers and stakeholders but also different societies, councils and colleges make sure that wellbeing on healthcare workers is considered. Leadership has been seen in countries like the United Kingdom where not only government issued the PPE guidelines but also different societies and the Royal Colleges stepped forward to provide updated guidelines, on regular basis [23,24]. Unfortunately, except for few local guidelines published recently by some hospitals, the same generalized response has not been witnessed in Pakistan. However, the Government alone released guidelines on PPE based on WHO-guidelines [3,7,25].

Many countries have been blamed for a slow response to tackle COVID-19, but because of different strains of the COVID-19 virus, the response has been different across the world [26]. In low-income countries like Pakistan, with limited resources and health system not as strong as developed countries, the response was also slower but mostly based on WHO guidelines. 65.2% of the participants in our survey were not happy with the preparations (Table 4). The response included strict to smart lockdown and education of the people using media [3]. In our survey most of the participants (83.6%) were favour of strict lock down, the reason of which may be multifactorial but were not accessed in this survey.

Since the time this survey went live in Pakistan on 15th April, the Government of Pakistan has brought many changes in the healthcare system to provide measures to help diagnose and contain the disease. Although the expectation has not been achieved, because of social beliefs and economic impacts, there has been a struggle to achieve the appropriate safety measures for the healthcare workers. Pakistan relies on the aid from within its rich population as well as from countries like China who are helping in providing equipment regularly to diagnose the disease and also for the protection of the healthcare workers. Relief Fund has been established by the Government of Pakistan to receive a donation for the welfare of the public. In different languages, social media services have been used for community education for preventive measures to decrease the number of the case so that healthcare workers are less exposed to the disease [27].

Despite many Standard Operating Procedures (SOPs), smart lockdown somehow did not work effectively in Pakistan which is evident by the regular increase in the number of cases, creating more risk of human-to-human exposure to healthcare workers and in-turn increasing the demand of PPE. Government of Pakistan, however, has recently been very successful in establishing designated isolation units and field hospitals across the country. Testing facilities have been made available more frequently recently and drive-through COVID-19 testing is also part of an initiative in Sindh, a province of Pakistan. However, due to panic frontline workers still hope to have COVID-19 tests freely available for them [3,21].

As frontline healthcare providers look towards the stakeholders with hope, smart strategies are required to ensure to continue to supply the PPE to the healthcare workers without disruption. Education and guidance of the healthcare workers are also important to understand who needs PPE more as the priority. Local industry can be reactivated and revitalized at the significant pace to help increase national PPE production and if possible regional cooperation.

This survey gives a general overview of the situation in a low-income country like Pakistan although most of the responses were from two main provinces only but still the results give a good broad overview of a low-income developing country.

## 5 Conclusion

The risks, drawn by COVID-19 in Pakistan, continue to create sense of insecurity due to lack of help from the leadership at all levels along with fear and panic. As world continues to learn, Colleges and Societies in the low-economic countries need to keep the pace with the world to come up with local policies and guidelines in accordance with WHO standards to help ascertain the wellbeing of their healthcare workers especially frontline warriors during this pandemic crisis.

## Ethical approval

As this survey focused on healthcare workers, no ethical approval required.

## Funding

None.

## Author contribution

Adeel Abbas Dhahri: Study design, data collection, data analysis, writing.

Muhammad Rafaih Iqbal: Writing, editing.

Abul Fazal Ali Khan: Editing.

## Registration of research studies

Name of the registry: Research Registry.

Unique Identifying number or registration ID: researchregistry5884.

Hyperlink to your specific registration (must be publicly accessible and will be checked): https://www.researchregistry.com/browse-the-registry#home/

## Guarantor

Adeel Abbas Dhahri

## Declaration of competing interest

None.

## Appendix A Supplementary data

Application Application
