# Supplementary material for: A cross-sectional survey on availability of facilities to healthcare workers in Pakistan during the COVID-19 pandemic
Source: Ann Med Surg (Lond). 2020 Sep 23;59:127–30. doi: 10.1016/j.amsu.2020.09.027 (PMC7510431; doi:10.1016/j.amsu.2020.09.027)
Supplement: Application [file mmc1.docx]

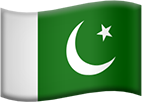
COVID-19 Pakistan survey

Dear colleague,

COVID-19 is spreading quite rapidly in Pakistan. Government of Pakistan is taking appropriate measures to slow the spread of the virus. We would be grateful if you can kindly fill out the survey below.

The main aim of this survey is to access the facilities available in Pakistan and understand the ground reality about the current Covid-19 pandemic.

All of your responses will be confidential.

Your participation is highly appreciated.

Many thanks

Regards

Adeel Abbas Dhahri

Muhammad Rafaih Iqbal

1. Which province do you work?
   - Punjab
   - Sindh
   - Baluchistan
   - Khyber Pakhtunkhwa
   - Gilgit Baltistan
2. Which hospital do you work?
   - Public sector
   - Private sector
   - Both
3. What is your role in the hospital?
   - Doctor
   - Nurse
   - Healthcare assistant
   - Manager
   - Pharmacist
   - Physiotherapist
   - Porter/ ward boy
   - Other
4. Do you have Standard Operating Procedure (SOPs) and policies regarding COVID-19 at your hospital?
   - Strongly agree
   - Agree
   - Neither agree nor disagree
   - Disagree
   - Strongly disagree
5. Do you have sufficient disposable face masks in your hospital?
   - Strongly agree
   - Agree
   - Neither agree nor disagree
   - Disagree
   - Strongly disagree
6. Is the availability of Personal Protective Equipment (PPE) adequate at your hospital? (PPE include gowns, gloves, respirator masks, eye protection)
   - Strongly agree
   - Agree
   - Neither agree nor disagree
   - Disagree
   - Strongly disagree
7. Have you been provided with Donning & Doffing training at your hospital?
   - Strongly agree
   - Agree
   - Neither agree nor disagree
   - Disagree
   - Strongly disagree
8. Do you have adequate specific isolation wards in your hospital for COVID-19 patients?
   - Strongly agree
   - Agree
   - Neither agree nor disagree
   - Disagree
   - Strongly disagree
9. Is your hospital management helpful to the employees in the current crisis?
   - Strongly agree
   - Agree
   - Neither agree nor disagree
   - Disagree
   - Strongly disagree
10. Where do you get most of the information regarding COVID-19?
    - Hospital emails/ messages/ notifications
    - NEWS
    - Social media
    - [www.covid.gov.pk](http://www.covid.gov.pk)
    - Friends and family
    - Health professionals
    - All of the above
11. Do you think Pakistan was appropriately prepared for this pandemic?
    - Strongly agree
    - Agree
    - Neither agree nor disagree
    - Disagree
    - Strongly disagree
12. Are you in favour of strict lockdown rather than smart lockdown?
    - Strongly agree
    - Agree
    - Neither agree nor disagree
    - Disagree
    - Strongly disagree
13. Has your hand washing improved in this pandemic?
    - Strongly agree
    - Agree
    - Neither agree nor disagree
    - Disagree
    - Strongly disagree
14. Do you think all the employees of your hospital should be tested for COVID-19?
    - Strongly agree
    - Agree
    - Neither agree nor disagree
    - Disagree
    - Strongly disagree
